# Extracting the invisible: obtaining high quality DNA is a challenging task in small arthropods

**DOI:** 10.7717/peerj.6753

**Published:** 2019-04-12

**Authors:** Andrea Lienhard, Sylvia Schäffer

**Affiliations:** Institute of Biology, University of Graz, Graz, Austria

**Keywords:** DNA extraction, Acari, DNA quantification, PCR, Comparison

## Abstract

**Background:**

The application of an appropriate extraction method is a relevant factor for the success of all molecular studies.

**Methods:**

Seven different DNA extraction methods suitable for high-throughput DNA sequencing with very small arthropods were compared by applying nine different protocols: three silica gel based spin methods, two cetyltrimethyl ammonium bromide (CTAB) based ones (one with an additional silica membrane), a protein precipitation method and a method based on a chelating resin (applying different protocols). The quantity (concentration) and quality (degradation, contamination, polymerase chain reaction (PCR) and sequencing success) of the extracted DNA as well as the costs, preparation times, user friendliness, and required supplies were compared across these methods. To assess the DNA quantity, two different DNA concentration measurements were applied. Additionally, the effect of varying amounts of starting material (different body sizes), variable lysis temperatures and mixing during DNA extraction was evaluated.

**Results:**

Although low DNA concentrations were measured for all methods, the results showed that—with the exception of two methods—the PCR success was 100%. However, other parameters show vast differences. The time taken to perform DNA extraction varied from 20 min to 2.5 h (Chelex vs. CTAB) and the costs from 0.02 to 3.46 € (Chelex vs. QIAamp kit) per sample. High quality genomic DNA was only gained from four methods. Results of DNA quantity measurements further indicated that some devices cannot deal with small amounts of DNA and show variant results.

**Discussion:**

In conclusion, using Chelex (chelating resin) turned out as a rapid, low-cost method which can provide high quality DNA for different kinds of molecular investigations.

## Introduction

For the success of all molecular investigations, such as DNA barcoding, metagenomic analysis by next-generation sequencing (NGS), etc., the DNA extraction is an important initial step. A variety of DNA extraction methods are available or have been used to isolate DNA from biological material. An optimal extraction method should optimize the DNA yield, avoid lab contamination and minimize degradation and inhibitors as well as costs, handling time, plastic consumables, equipment, toxic materials etc. (Queipo-Ortuno et al., 2008; Chen et al., 2010; Knebelsberger & Stöger, 2012). In the last few years, sequencing technologies have undergone major improvements and NGS is about to be integrated in routine practice. Nevertheless, studies focusing on several DNA extraction methods designed for subsequent NGS approaches are scarce. Five common methods on fecal samples (Hart et al., 2015), three methods for high-throughput DNA sequencing with ancient horse DNA (Gamba et al., 2016), six commercial kits to extract bacterial DNA (Becker et al., 2016), and eight extraction techniques for species comprising eight different phyla (Schiebelhut et al., 2017) were, for example, compared in recent studies.

A very important and limiting factor in processing molecular investigations is the usually small amount of suitable starting DNA (often in adequate quality). Low DNA quantities can be the result of working with highly degraded samples (e.g., museum specimens, fossils, forensic samples) or of small amounts of starting material (by extracting, e.g., very small specimens or only parts of specimens). A few examples of studies that compare different DNA extraction methods in small arthropods are available, although only two dealt with single mite individuals in comparable size to the herein used study organisms (oribatid mites): four methods for the mite species *Dermanyssus gallinae* (Desloire et al., 2006) and three methods investigating an oribatid mite species (Per & Ercan, 2015). The purity and the quantity of the extracted DNA as well as the PCR success were evaluated, but other important factors like the successful gain of high molecular weight DNA were not taken into account in these studies (Desloire et al., 2006; Per & Ercan, 2015). However, the quality of the extracted DNA is important for reliable results (the number of potential SNPs decreases with decreasing quality) in NGS applications (Graham et al., 2015). As Carew, Coleman & Hoffmann (2018) stated, particular extraction methods perform better for some taxa in terms of the number of reads (Illumina MiSeq) and the number of positively sequenced barcodes for invertebrate samples. The purity of DNA is a further important factor in gaining reliable results for NGS and has, for example, an effect on the reproducibility of library preparation for Illumina sequencing (Lamble et al., 2013; Arseneau, Steeves & Laflamme, 2017).

Oribatid mites are small arthropods which play an important role in the decomposition of organic substances in all parts of the world (Norton & Behan-Pelletier, 2009). The small size of these organisms (body size ranges from 0.2 to 1.5 mm) indicates a low amount of starting material; hence, whole specimens have to be extracted for high DNA quantities. With the extraction of whole specimens, contaminants and inhibiting substances are unavoidable and remain in the extraction process. Moreover, oribatid mites are heavily sclerotized, making it difficult for chemicals to enter tissue for the lysis (thus, specimens have to be mechanically damaged).

Five commercially available DNA extraction kits and two traditional DNA extraction methods were tested in this study. All DNA extraction kits are spin procedures using columns equipped with a silica membrane. The two traditional extraction methods are a protein precipitation method (cetyltrimethyl ammonium bromide; CTAB) and a chelating resin (Chelex), respectively. Common quantification methods were used to assess the quality as well as the quantity of the obtained DNA. The DNA quantity was measured by means of two different concentration measurement methods: (i) absorbance at 260–280 nm with a UV spectrometer, (ii) fluorescent dye that binds to DNA. The quality of the DNA (or PCR products) was evaluated by means of three different techniques: via the assessment of band intensities and lengths in an agarose gel electrophoresis, by means of a gel electrophoresis with an automated analyzing instrument, and by assessing the PCR and sequencing success. The effect of different lysis temperatures and mixing on the degradation of DNA during extraction was also investigated. As the condition of the source material plays an important role concerning the quality and quantity of the extracted DNA, individuals varying in body size (small, average and large) were investigated for each method and two chemicals for DNA preservation were tested (absolute ethanol and propylene glycol). Moreover, the costs, handling time, user friendliness, the use of toxic chemicals and the generation of waste were taken into account.

Studies dealing with “small” arthropods obtained different findings, however, in most previous studies silica gel based spin methods gave the best results (Chen et al., 2010; Dittrich-Schröder et al., 2012; Athanasio et al., 2016; Kranzfelder, Ekrem & Stur, 2016). So far no study was conducted dealing with very small arthropods (<1 mm) comparing several important factors across different extraction methods with the aim of gaining high quality DNA for NGS approaches.

This study should provide a guide for gaining high quality DNA and for choosing the DNA extraction method most suitable for very small arthropods, considering several important factors like DNA quantity and purity, costs, handling time, PCR success, etc.

## Materials and Methods

For each method, single individuals of three oribatid mite species, which differ in their relative size (body length) were extracted: *Tectocepheus* sp. (300–350 µm), *Paraleius leontonychus* (440–500 µm) and *Hermannia* sp. (800–950 µm).

Specimens were preserved in either absolute ethanol or propylene glycol for several weeks. A total of 24 h prior to DNA-extraction, propylene glycol stored material was transferred into absolute ethanol. For all extraction methods whole individuals were crushed against the tube wall with a metal brad and total genomic DNA (gDNA) was isolated.

### DNA extraction methods

Seven different DNA extraction methods (nine different protocols) were applied. As RNase treatment is said to be an important step for gaining pure, high quality DNA, it was added to all methods. However, in three Chelex protocols (CH4–6; see below) the RNase step was omitted, because we were particularly interested in the effect of temperature and/or mixing on the quality of DNA alone.

1. NucleoSpin Tissue XS (=NST; Macherey-Nagel, Düren, Germany): protocol followed the manufacturer’s instructions; additionally, 2 µl RNase (5 ng/µl) was added and incubated for 15 min (22 °C, 450 rpm) after the first lyse step. Samples were incubated for 4 h and eluted in a volume of 25 µl.
2. QIAamp DNA Mini kit (=QIA; Qiagen, Hilden, Germany): extraction followed manufacturer’s instructions applying one elution step with 50 µl buffer.
3. PeqGOLD Tissue DNA Mini kit (=PEQ, s-line; peqlab, VWR, Erlangen, Germany): all liquids were reduced by 50%; Samples were eluted in a volume of 50 µl.
4. Wizard gDNA kit (=WIP; Promega, Madison, WI, USA): we applied the “animal tissue (mouse tail)” protocol, again with the modification of reducing all substances by half. In case of the “ethanol aspirate step” the liquid was evaporated in a vacuum centrifuge rather than simple aspiration. For the last step 30 µl rehydration solution was added.
5. CTAB (=CTAB): standard protocol (Schäffer et al., 2010) was adapted by adding 3 µl RNase after the lysis step (incubation at 22 °C for 15 min, 450 rpm); elution volume was 25 µl.
6. E.Z.N.A.® Insect DNA kit (=EZNA; Omega Bio-tek, Inc., Norcross, GA, USA): protocol followed the manufacturer with a final elution volume of 75 µl.
7. Chelex (=CH): this method was conducted by applying three standard protocols (CH1–CH3): 5 mg Chelex 100 resin were incubated in 50 µl ddH_2_O at (i) 95 °C for 20 min without any further treatment (CH1), (ii) 95 °C for 20 min followed by a RNase treatment (using 5 ng/µl RNase at 37 °C for 8 min) for the removal of RNA (CH2) and (iii) 56 °C overnight, including 2 µl Proteinase K (with final enzyme inactivation at 95 °C for 10 min), also followed by a RNase step (CH3).

All kit extracted samples were eluted with the provided elution buffers (methods 1–4, 6). For the CTAB method a lab prepared TE-buffer was used. These buffers were also used as blank samples for the subsequent quality and quantity measurements. A list of all applied DNA extraction methods with incubation times is given in Table 1.

**Table 1:** List of applied DNA extraction methods, with the costs per sample, preparation and lysis time.

DNA extraction method	Company	Method	Cost per sample^1^ (€)	Not included^2^	Preparation time^3^ (min)	Lysis time (h)	Source
NucleoSpin Tissue XS (NST)	Macherey-Nagel	Silica membrane with spin columns	3.12	Ethanol 2 × 8 tubes RNase	40–45	3–4	http://www.mn-net.com/Products/DNAandRNAPurification/DNAfromtissueandcells/NucleoSpinTissueXS/tabid/10645/language/en-US/Default.aspx
QIAamp DNA Mini kit (QIA)	Qiagen	Silica membrane with spin columns	3.46	Ethanol 2 × 8 tubes RNase	50	3	https://www.qiagen.com/at/shop/sample-technologies/dna/genomic-dna/qiaamp-dna-mini-kit/#orderinginformation
PeqGOLD Tissue DNA Mini kit (PEQ)	peqlab	Silica membrane with columns	1.41^4^	Ethanol 3 × 8 tubes	40–45	3	https://de.vwr.com/store/product/16763411/tissue-dna-mini-kits-peqgold
Wizard Genomic DNA kit (WIP)	Promega	Protein precipitation method	1.68^5^	70% Ethanol, Isopropanol 3 × 8 tubes	55	3	https://www.promega.com/products/dna-purification-quantitation/genomic-dna-purification/wizard-genomic-dna-purification-kit/
CTAB	SIGMA	Cetyltrimethyl ammonium bromide, 2 phase-chloroform precipitation method	1.00^6^ 0.6–0.8^7^	NaCl, EDTA, TrisHCl, Proteinase K, β-mercaptoethanol, Chloroform, Isopropanol, Ethanol, TE-buffer 3 × 8 tubes RNase	135^8^	3	http://www.sigmaaldrich.com/catalog/product/sigma/h6269?lang=de&region=AT
E.Z.N.A. Insect DNA kit (EZNA)	Omega Bio-tek, Inc.	Cetyltrimethyl ammonium bromide based method with a silica membrane with spin columns	1.72	Ethanol, Isopropanol, Chloroform, Isoamyl alcohol 3 × 8 tubes	80	4	https://at.vwr.com/store/product/10925134/e-z-n-a-insect-dna-kit
CHELEX (CH)	Bio-Rad	Chelating ion exchange method	0.02	Water 8 tubes RNase (Proteinase K)	1.5	0.20	http://www.bio-rad.com/de-at/sku/1421253-chelex-100-resin

**Notes:**

1 list price for 50 samples, not included substances (for the commercially available kits) and disposables (additional tubes, pipette tips) were excluded; for CTAB all substances listed in table were included in calculation; Chelex was calculated for 50 g.

2 for all methods manual pipettors with disposable tips, a centrifuge, a vortex mixer and a thermal heating-block are required; for CTAB and the EZNA a fume hood is required; for the WIP a vacuum centrifuge was used.

3 for eight samples excl. lysis and RNase incubation steps, incl. waiting steps (centrifugation, cooling, etc.).

4 calculated with a 50% reduced volume and the price of 25 columns was added.

5 calculated with a 50% reduced volume.

6 Zetzsche et al. (2008).

7 Chen et al. (2010) (including microcentrifuge tubes and pipette tips) and Kranzfelder, Ekrem & Stur (2016).

8 Isopropanol precipitation step (overnight) and time spent for buffer preparation was excluded.

Finally, to evaluate the effect of temperature and mixing in the Chelex procedure on the quality of DNA, the following three approaches were evaluated: (1) Chelex 4 (CH4): without the 95 °C Proteinase K inactivation step, but with a 10 s mixing step after the incubation; (2) Chelex 5 (CH5): without vortexing, but with the inactivation step at 95 °C for 10 min; (3) Chelex 6 (CH6): without enzyme inactivation and vortexing. In all approaches (CH4–CH6) samples were incubated at 56 °C for 4 h with 2 µl Proteinase K. An overview of the different Chelex protocols is given in Table S1.

### DNA quantity and quality

To estimate the concentration of DNA, the Promega Quantus Fluorometer and the Implen NanoPhotometer® were utilized. Measurements with the Promega Quantus Fluorometer were evaluated with the QuantiFluor dsDNA Dye which is a fluorescent DNA-binding dye enabling sensitive quantitation of small amounts of double-stranded DNA in solution. With the Implen NanoPhotometer® NanoDrop the DNA concentration as well as the purity of the DNA (A260/280) was assessed; whereby absorption at 260 nm indicated the presence of DNA and at 280 nm the presence of proteins, phenol or other contaminants.

The Agilent 2200 TapeStation with the Agilent gDNA ScreenTape assay was used to assess the quantity and integrity of the gDNA in the samples. In a first step quality measurements were taken from single individual extracts. However, as our samples are below the required DNA concentration of 10–100 ng/µl, no bands were visible. Therefore, three *Hermannia* and six *Paraleius* individuals were pooled and extracted applying the same protocols as described above, except for an increased elution volume (NST and the CTAB method 30 µl). The integrity of gDNA was determined with the TapeStation Analysis Software and is given as the DNA integrity number (DIN) indicating the fragmentation of the gDNA on a scale from 1 (highly intact DNA) to 10 (strongly degraded DNA).

### PCR and sequencing

The D3 region of the nuclear 28S rRNA (using the primer pair D3A and D3B; Litvaitis et al., 1994) was amplified according to the standard protocol (Schäffer et al., 2008). Subsequently, PCR products (two µl) were loaded on a 2% agarose gel to verify the success of PCR amplification. PCR conditions, purification and Sanger sequencing steps were conducted as described by Schäffer et al. (2008) and Koblmüller, Salzburger & Sturmbauer (2004) on an automated capillary sequencer (ABI PRISM 3130xl). Sequencing was performed in both directions. All sequences were verified by comparisons with previously published oribatid mite sequences at GenBank.

## Results

### Costs, required substances and consumables, handling time and user friendliness

Methods 1–3 (QIA, PEQ, NST)—The preparation times and the cost only slightly vary among the three tested silica membrane methods (Table 1). An advantage of these kits is that the purification requires no toxic phenol/chloroform steps or isopropanol/alcohol precipitation. Thus, the commercial kits do not generate hazardous waste or do not require a fume hood to operate. All silica membrane kits only require the supply of ethanol. However, the need of plastic consumables is relatively high.

Method 4 (WIP)—This method is slightly less expensive than the three silica membrane based kits mentioned before, mainly because the volume was reduced by 50% for all steps. For this kit no tubes were provided.

Methods 5 and 6—CTAB is the most time consuming method, requiring many different steps. Moreover, toxic (or harmful) substances, such as β-mercaptoethanol and chloroform (carcinogen), which require a fume hood, have to be used. Also for the EZNA protocol (based on the CTAB method), the use of toxic substances (chloroform) is required and many different pipetting steps (phase separations) have to be conducted. The high laboratory costs were not included in the calculation (because they will vary from lab to lab, depending on the salary of the employee performing the work) and would further be a disadvantage of both CTAB based methods.

Method 7 (Chelex)—this method is the most cost and time efficient approach tested in this study; the preparation time for eight samples is just a few minutes and the costs are far below 0.10 € per sample. The three silica-membrane based kits (mean of 3.13 €) have increased costs per sample by a factor of approximately 150, compared to the Chelex method (0.02 €). Due to the fact that the total reaction occurs in one tube and there is just one pipetting step necessary (or two to three by adding Proteinase K and/or RNase), the waste of disposables (tubes, pipette tips) is also very low.

### DNA quantity and quality

In the majority of cases, the quantity values obtained by the NanoDrop Photometer were much higher than those of the QuantiFluor. Due to the fact, that we measured at the detection limit of the device, all quantity values as well as the purity measurements (variation among samples within a particular treatment was high) are questionable. Nonetheless, for comparison NanoDrop values are given in Table S2 and Table S3.

Concerning the Quantus Fluorometer measurements, the highest mean DNA quantity in ng/µl was obtained applying the Chelex method at 95 °C without RNase treatment (CH1, mean value of all specimens 0.39 ng/µl) followed by the QIA (0.34 ng/µl). The lowest values were measured for the PEQ and the WIP (0.03 ng/µl). Moreover, three DNA extracts of the WIP showed a value lower than the blank (Table 2). CH1 (95 °C, without RNase) yielded the highest concentration of a single extracted specimen (1.5 ng/µl, *Hermannia* sp.). Furthermore, the propylene glycol preserved specimens showed on average higher DNA concentrations (0.07 vs. 0.08). As elution volumes differed between the investigated methods, quantity values were standardized to 100 µl volume.

**Table 2:** Concentration measurements (ng/μL) of DNA extracts by fluorometry (QuantiFluor), obtained from three oribatid mite species.

Extraction method	*Tectocepheus* sp.		*Paraleius leontonychus*		*Hermannia* sp.	
NST (25 µl)	0.003	0.002	0.147	0.329*	0.020	0.015
QIA (50 µl)	0.053	0.016*	0.115	0.135*	0.969	0.770
PEQ (50 µl)	0.005	0.007^3^	0.014	0.048	0.096	0.039
WIP (30 µl)	x^1^	x^1^*	x^1^	0.002*	0.176	0.015
CTAB (25 µl)	0.041	0.031	0.102	0.039*	0.006	0.021
EZNA (75 µl)	0.021	0.015	0.063	0.071*	0.696	–
CH1 (50 µl)	0.054	0.081*	0.115	0.095*	0.506	1.460
CH2 (50 µl)	0.024	0.014	0.040	0.048*	0.217	0.285
CH3 (50 µl)	0.016	0.023	0.027	0.053*	0.288	0.332

**Notes:**

Same samples per species are indicated by same shades (white or gray) as found in Table S2. Values are given for single individuals (two per species). For the EZNA only one *Hermannia* individual was investigated. Values based on one measurement (control measurements revealed no deviating values). Elution volumes are given in parenthesis.

x = value lower than blank.

1 no PCR product.

* specimens preserved in propylene glycol.

**Figure 1:**
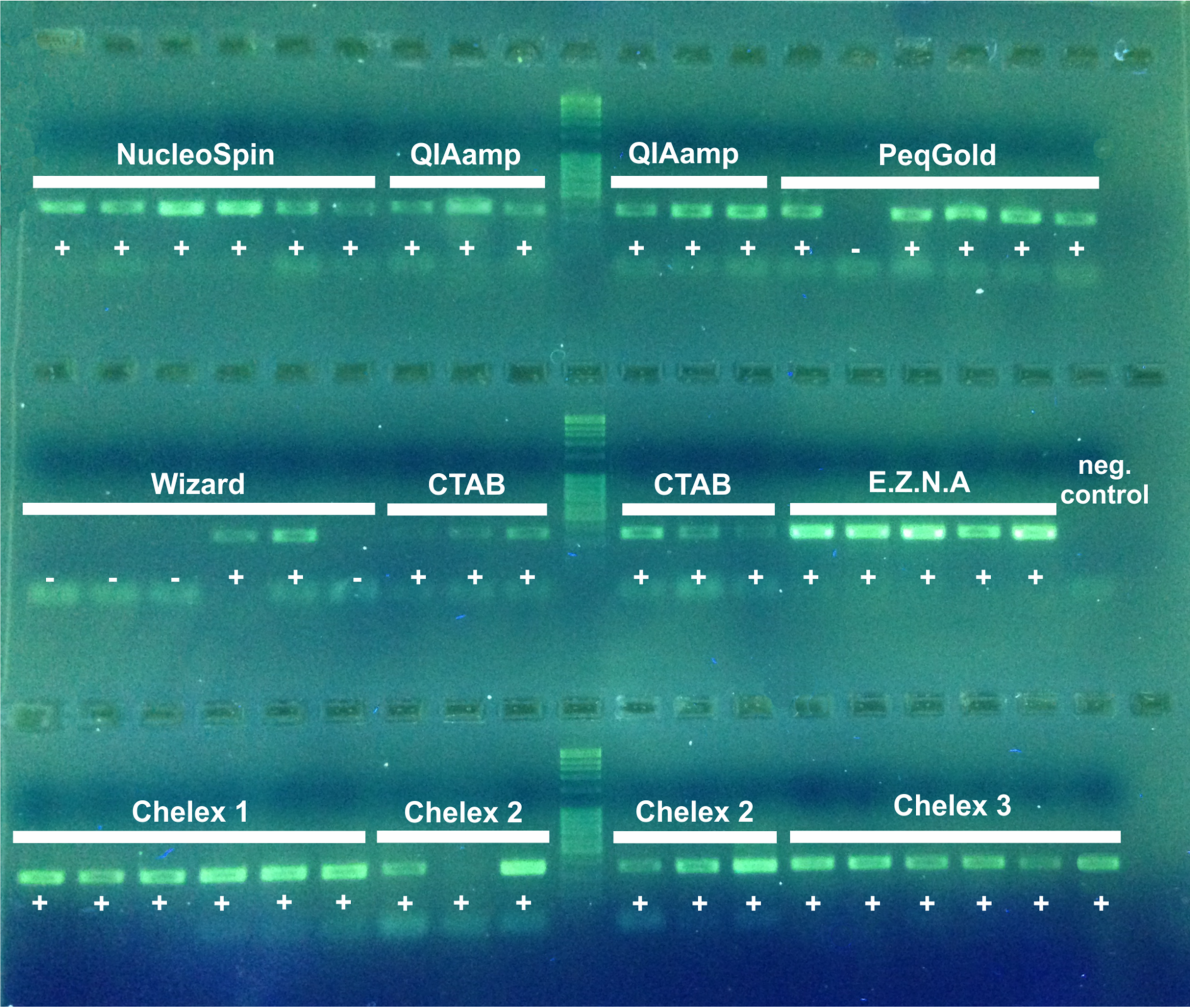
2% agarose electrophoresis gel of PCR products (D3) of nine DNA extraction methods. Sequencing success is indicated by a +, failure −.

A sharp and intense high molecular weight band (at approximately 48.5 kbp) indicating intact gDNA with minimal degradation is only present in *Hermannia* samples extracted with the following methods/protocols: NST, QIA, EZNA, CH4 and CH6 (see Fig. 1). For all other samples only degraded DNA—visible as a smear in the lower molecular weight part of the gel—is present. Only Chelex protocols without the heat incubation step (at 95 °C) provided less degenerated DNA, the vortexing step (10 s), however, had only a small effect on the degradation of the DNA. Since DNA concentrations were very low, DIN values could not be calculated.

### PCR and sequencing success

**Figure 2:**
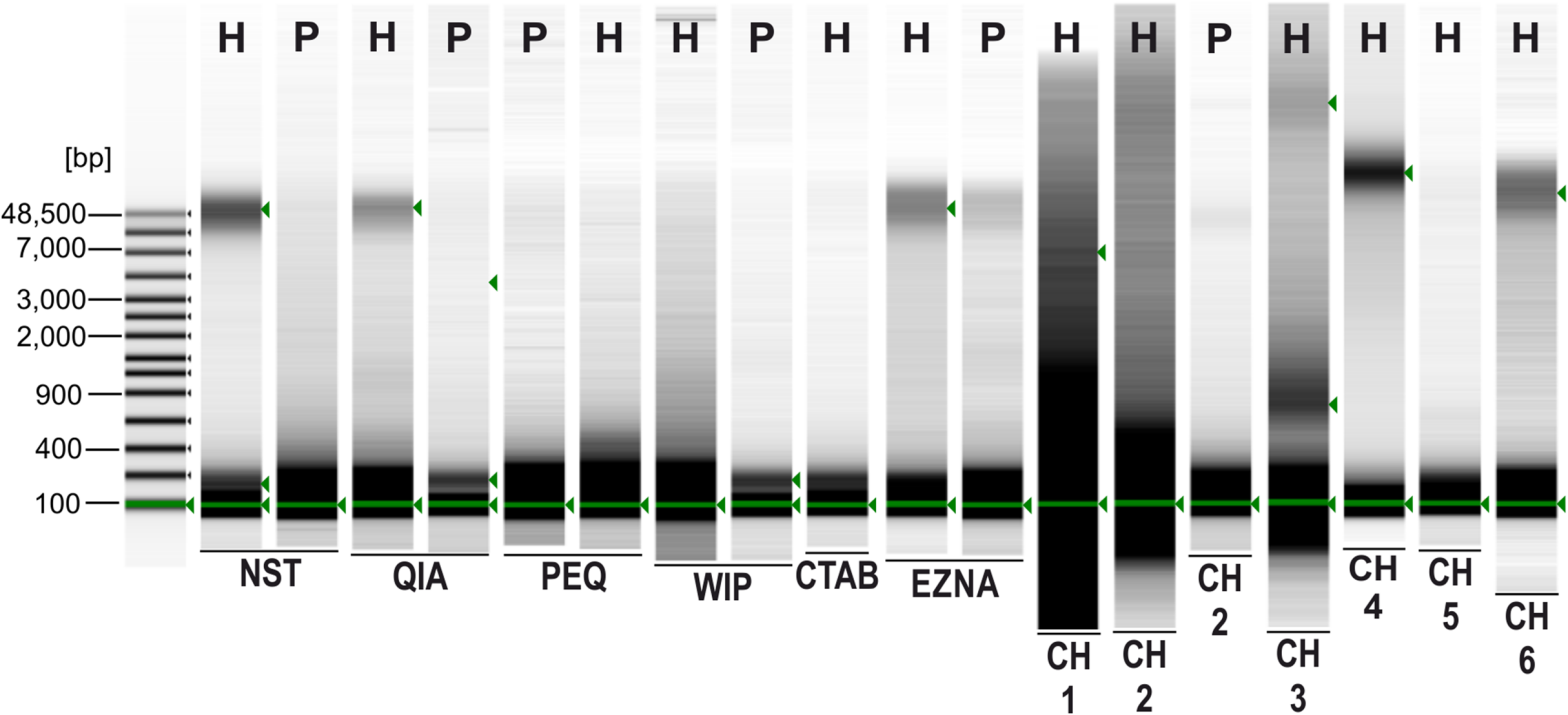
Agilent 2200 TapeStation gel image obtained from seven extraction methods. H, *Hermannia* with three individuals pooled; P, *Paraleius* with six individuals pooled.

PCR success was indicated by the presence of a band for each sample on an electrophoresis gel (Fig. 2). NST, QIA, EZNA, CTAB and all Chelex extractions show a 100% PCR amplification success (for Chelex one slight band was only visible by eye). The PEQ had moderate PCR success with 83%, and for the WIP only 33% of the samples showed a positive amplification. All samples showing a positive amplification on the agarose gel were sequenced. Sequencing of the 311–317 bp fragment confirmed that all PCR products were from our target organisms. No overlays (multiple peaks) or degradation of DNA was detected in the obtained fragments. All sequences are available at GenBank (accession numbers MK543124–MK543172).

## Discussion

### Comparison of the extraction methods

The first three extraction methods tested use a silica membrane with spin columns (methods 1–3). All these spin procedures are based on the same principle and comprise the same four steps: lysis, binding, washing and elution. All protocols are very easy to perform using standard equipment commonly available in most laboratories. The WIP (method 4) is a protein precipitation method based on four main steps: lysis and protein precipitation, RNase digestion, DNA salt and isopropanol precipitation and a rehydration step. This method is more complicated to handle because of several supernatant pipetting steps. For the last alcohol discarding step a special pipette (with narrow tips)—which was not available in our lab—is recommended to avoid aspiration of the very loose DNA pellet into the pipette. As in our case pellets are generally too small to be visible to the eye, we removed the liquid in the vacuum centrifuge rather than with a pipette. The next two methods are based on a cationic detergent, namely CTAB. The CTAB protocol with the laboratory prepared buffer comprises a lyse step, a CTAB/chloroform phase segregation, an isopropanol precipitation, two ethanol washing steps and a dry-resuspending step. As with the WIP, the CTAB method is difficult to handle because of several supernatant pipetting steps, which demand pipetting experience (especially because of the hardly visible pellet). The EZNA was especially designed for the recovery of gDNA from arthropods. The procedure relies on the CTAB method, in conjunction with the selective DNA binding of Omega Bio-tek’s HiBind DNA column to isolate DNA. After the lysis, a chloroform-isoamyl alcohol step with a subsequent phase separation has to be performed. Then, ethanol should be added and the samples have to be loaded on the provided columns. A wash and an elution step complete the extraction. Although the phase segregation requires pipetting experience, the handling is easier than for the WIP and CTAB, because the DNA is bound to the membrane in the column and is not present as pellet. Chelex is a chelating ion-exchange resin that binds polar components of cells leading to cell lysis. The remaining non-polar DNA remains in the aqueous solution above the Chelex. This resin prevents DNA degradation by binding (chelating) metal ions (Mg^2+^) that catalyze the breakdown of DNA. The comparison of seven different extraction methods showed that the Chelex method is the cheapest, fastest and the easiest to handle approach tested in this study. Moreover, in contrast to other methods, it is ecologically friendly with no toxic chemicals and the reduced plastic waste (Table 3). Furthermore, Chelex yielded the largest amount of DNA. Besides the generally higher costs, the silica based spin methods NST and QIA showed a moderate “eco-friendliness,” while the EZNA kit had an overall poor performance regarding handling time, user friendliness and the consumption of plastic and hazardous waste.

**Table 3:** Performance of 12 different DNA extraction protocols.

	PCR and sequencing success	Quality of DNA			Quantity of DNA^1^	Purity of DNA	Costs	Handling time	User friendliness	Plastic waste	Hazardous waste
NST	+	+			∼	−	−	+	+	∼	+
QIA	+	+			++	−	−	+	+	∼	+
PEQ	∼	−			∼	*	∼	+	+	−	+
WIP	−	−			∼	−	∼	+	∼	−	+
EZNA	+	+			+	−	∼	−	−	−	−
CTAB	+	−			∼	−	+	−	−	−	−
CH1	+	−			++	+	++	++	++	++	+
CH2	+	−			+	∼	++	++	++	+	+
CH3	+	∼			+	−	++	++	++	+	+
CH4–CH6	+	+^a^	−^b^	+^c^	+	*	++	++	++	+	+

**Notes:**

++ = very good, + = good, ∼ = moderate, − = negative; these symbols are based on author’s personal opinion and experiences.

1 based on measurements with the Quantus Fluorometer.

a CH4.

b CH5.

c CH6.

* not measured.

Previous comparisons of DNA extraction methods evaluated in the present study revealed variable results, but in most of the studies silica-membrane based methods provided the best results for the isolation of DNA from small arthropods, like wasps, diptera exuviae or mites (Zetzsche et al., 2008; Dittrich-Schröder et al., 2012; Ammazzalorso et al., 2015; Kranzfelder, Ekrem & Stur, 2016). Even though the performance of extraction methods with “small” arthropods was examined in several studies (beetles ∼5 mm, Chen et al., 2010; minute hymenopterans, adulti ∼1 mm, Dittrich-Schröder et al., 2012; daphnia, up to 6 mm, Athanasio et al., 2016; diptera exuviae, a few mm, Kranzfelder, Ekrem & Stur, 2016; Chironomidae, adulti usually less than 1 mm, Wang & Wang, 2012), all organisms exceed body length of our study species by up to 20-fold. Thus, none of these mentioned studies—which compare different extraction methods—investigated specimens of comparable size to our study organisms. However, there are two investigations dealing with individual mites (in comparable size) and the comparison of different extraction methods (Desloire et al., 2006; Per & Ercan, 2015). For the mite species *D. gallinae* CTAB, Chelex, a QIAamp DNA extraction kit and a filter-based technology method were examined with the conclusion that Chelex was the least efficient in terms of DNA amplification, whereas the best results were obtained using the CTAB method and the QIAamp kit. By investigating the oribatid mite species *Parachipteria willmanni* three techniques (Chelex, a Qiagen extraction kit and a CTAB method) were compared (Per & Ercan, 2015). Congruent with our results, the Chelex method yielded the largest amount of DNA, but protein contamination was very high (the values are also questionable because of the detection limit of the used NanoDrop).

As NGS offers great new opportunities, the initial step of this analysis—which is the extraction of DNA—should be optimized. It has been shown, that the specific method of DNA extraction used has a strong impact on the number of successfully amplified samples for NGS. The performance varies depending on the study species (Hart et al., 2015; Carew, Coleman & Hoffmann, 2018), and commercial silica-based methods are more advantageous than in solution methods providing more complex DNA libraries. Carew, Coleman & Hoffmann (2018) showed the importance of sufficient starting material for MiSeq reads. Whole specimens of beetles produced >1,800 reads, if only legs were used <20 reads were produced. Another example showed that six tested commercial extraction kits (including the WIP) yielded satisfactory MiSeq results (Gamba et al., 2016). Nevertheless, factors like costs or preparation time were not considered (Gamba et al., 2016). In general, commercial extraction kits were used for gDNA extraction in varying NGS approaches of minute arthropods, as copepods (Blanco-Bercial & Bucklin, 2016), microhymenoptera (Cruaud et al., 2018) and also mites (Dong et al., 2018; Edwards et al., 2018; Esteban et al., 2018; Lee & Wang, 2016; Schäffer et al., 2018; Techer et al., 2019).

### DNA quantity and quality

With respect to the small size of our study species, DNA quantity was generally low in all specimens. Only measurements of the Quantus Fluorometer revealed reliable results (Table 2), however, comparing data from literature on DNA quantity of such small organisms in general are completely missing. Furthermore, the influence of RNase, different amounts of starting material and different sample preservations on DNA quantity should be addressed. Comparing the Chelex protocols with and without RNase treatment, the concentration was always higher without an RNase step (on average four times higher); this is probably related to the high temperature needed in the inactivation step. Moreover, a positive linear relationship between body size and DNA concentration could be detected (Fig. S1), as extracts of larger mite individuals provided higher DNA concentrations than smaller ones—a result also obtained in investigations on spiders (Casquet, Thebaud & Gillespie, 2012) and other mites (Tixier et al., 2010). Regarding sample preservation and storage, we observed on average higher DNA concentrations from propylene glycol preserved material compared to ethanol stored one (although high variance in DNA concentrations obtained by the same DNA extraction method occurred). A similar effect (propylene glycol was significantly better for high quality DNA than the storage in ethanol) was detected for other arachnid DNA samples too (Vink et al., 2005). Another useful argument in this context is the fact that propylene glycol can be used as a capture medium in pitfall traps.

Nonetheless, regardless of the quantity, nearly all tested methods are able to provide sufficient DNA for standard PCR amplifications and the subsequent sequencing process (Fig. 2; Table 3). Moreover, the DNA purity is another important feature to obtain PCR products. Although several authors noted that proteinases used for cell lysis may inactivate the Taq polymerase (if DNA purification is inadequate, Wilson, 1997), affecting PCR amplification negatively, the non-inactivation of Proteinase K in the herein tested CH6 protocol had no observable effect on the PCR success (see also comparison with CH4 and CH5 with included inactivation step, Fig. S2). Other inhibiting PCR factors can originate from the used extraction reagents or the investigated specimens themselves, for example CTAB, ethanol, fat, protein, isopropanol, phenol etc. (Demeke & Jenkins, 2010). In the present study, whole individuals containing amongst other things high amounts of proteins were used for the lysis. Remaining proteins present in the DNA solution can tightly bound to DNA and may interfere with subsequent DNA extraction steps or the PCR (Suárez-Martínez, Gonzalez-Santos & Montealegre, 2006). Considering the employed Chelex methods, there was no negative impact on PCR performance, although no purification steps were carried out and body remnants of the whole individual remained in the solution.

Whereas the fragment length, respectively, the quality of the DNA, is generally not that crucial for standard PCRs, certain NGS approaches require high molecular gDNA as starting material for the library preparation. For the application of restriction site associated NGS (RADSeq) Graham et al. (2015) exemplarily detected large-scale loss of Illumina reads caused by in situ DNA degradation. Moreover, high quality DNA can also be important for “standard PCR techniques,” especially if a long term storage is needed to conserve genetic resources, as for example, in barcoding studies (Knebelsberger & Stöger, 2012). In the framework of the present study, high molecular DNA could be successfully extracted, besides three commercial kits (NST, QIA, EZNA), by means of two Chelex protocols (CH4, CH6). In this context, it became apparent that the quality and quantity of extracted DNA may be affected by the lysis temperature. Heat treated Chelex samples (CH1–3, CH5) no longer contained intact, high molecular weight DNA demonstrating significant levels of DNA degradation. This physical treatment (exposure to heat) causes random breaks in the DNA strands, reducing fragment size and hence producing large amounts of low molecular weight DNA (Steiner et al., 1995). For all other methods the suggested incubation temperatures slightly varied; from 50 °C (PEQ) to 60 °C (EZNA). As with 3–4 h the lysis time is congruent among all tested methods, it appears to be not crucial for recovering intact DNA (at least in our study). In this context, Shahjahan et al. (1995) demonstrated that a lower incubation temperature (37 °C) resulted in more than double the amount of total DNA and the lowest mean absorbance ratio compared to incubations at 80 °C applying the CTAB method. Thus, the use of lower incubation temperatures can be recommended.

### The Chelex method

Considering all criteria in this study, the Chelex method had the overall best performance. It is a well-established method and is mainly applied in genetic studies on vertebrates (Estoup, 1996). If this technique was used in small organisms, several individuals were usually pooled together in one extraction. However, extracting single mite individuals is also possible with the Chelex method, as has been shown for, for example, the itch mite *Sarcoptes scabiei* (Skerratt et al., 2002), the spider mite *Brevipalpus phoenicis* (Groot et al., 2005), or the predatory mite *Neoseiulus baraki* (Sourassou et al., 2012). Although the Chelex method based on the protocol of Walsh, Metzger & Higuchi (1991) was also successfully used with single oribatid mite specimens about 20 years ago (Perrot-Minnot & Norton, 1997), further examples investigating Oribatida are rare in literature (Pepato, Da Rocha & Dunlop, 2010; Per & Ercan, 2015).

One common problem using Chelex is the assumption that Chelex extracts are not stable and suitable for long-term conservation. By means of classic Chelex extraction protocols, single stranded DNA is obtained instead of double stranded one (Casquet, Thebaud & Gillespie, 2012) with the result that the classic method cannot produce high purity DNA suitable for long-term storage (Hajibabaei et al., 2005). A modified Chelex extraction protocol (without a boiling step, yielding double-stranded DNA)—referred to as CH4 and CH6 in our study –that was tested on small spiders, however, was shown to be suitable for long term storage, with successful PCRs performed ∼1.5 years after extraction and several freeze-thaw-cycles (including two thawing events over 48 h) (Casquet, Thebaud & Gillespie, 2012). In general, there are clearly quite a few points that argue for the use of Chelex: the method is (i) inexpensive, (ii) harmless (and substances are readily available), (iii) simple (one step), (vi) quick, (v) providing a good quantity of DNA (also with small amounts of tissue) and (vi) suitable for long-term storage (over 1.5 years, because of the obtained double-stranded DNA) (Casquet, Thebaud & Gillespie, 2012). A possible limitation of the Chelex method is that the DNA is not purified prior to PCR, which may lead to PCR failure caused by potential inhibitors that remain in the extract (Casquet, Thebaud & Gillespie, 2012), something that has not been observed in our study.

## Conclusions

Every method has certain advantages and disadvantages and each scientist has to find the method most suitable for the specific study and/or organism. Considering all criteria described in the present study, the Chelex method without any heat treatment (CH4 and CH6) had the best overall performance and provided high quality DNA potentially useful for a broad range of genetic applications. The herein presented findings are likely applicable to other very small invertebrates too. However, there is no “gold standard” for DNA extraction and all important criteria should be considered and balanced for the specific aim of a study.

## Supplemental Information

10.7717/peerj.6753/supp-1 Supplemental Information 1 Differences between applied Chelex protocols (CH1–CH6).

10.7717/peerj.6753/supp-2 Supplemental Information 2 Concentration measurements (ng/µL) of DNA extracts by UV spectrometry (NanoDrop), obtained from three oribatid mite species.

10.7717/peerj.6753/supp-3 Supplemental Information 3 Purity of DNA extracts (mean OD values for each method, highest and lowest value, SD and number of measured samples) obtained from nine different methods.

10.7717/peerj.6753/supp-4 Supplemental Information 4 Relationship between body size (small, medium, large) and mean DNA concentration (ng/µL).

10.7717/peerj.6753/supp-5 Supplemental Information 5 2% agarose electrophoresis gel of PCR products (D3) of three Chelex extraction protocols (CH4–CH6).

10.7717/peerj.6753/supp-6 Supplemental Information 6 Original image of TapeStation Part1. This image was combined with image Part2 in Fig. 2 of the manuscript. Not all lanes are used for the present study.

10.7717/peerj.6753/supp-7 Supplemental Information 7 Original image of TapeStation Part2.

10.7717/peerj.6753/supp-8 Supplemental Information 8 Fasta file of 49 sequences.

## Additional Information and Declarations

### DNA Deposition

The following information was supplied regarding the deposition of DNA sequences: A FASTA file of DNA sequences is available as a Supplemental File. DNA sequences are provided at Genbank, accessible under GenBank accession numbers: MK543124–MK543172.

### Data Availability

The following information was supplied regarding data availability: The raw data are available in the Supplemental File.
