# Supplementary material for: Extracting the invisible: obtaining high quality DNA is a challenging task in small arthropods
Source: PeerJ. 2019 Apr 12;7:e6753. doi: 10.7717/peerj.6753 (PMC6463856; doi:10.7717/peerj.6753)
Supplement: Supplemental Information 1 [file peerj-07-6753-s001.docx]

**Differences between applied Chelex protocols (CH1-CH6).**

| Chelex Protocol | Incubation  Temp. [°C] | Incubation  Time | Proteinase  K | Proteinase K  inactivation^1^ | Vortex | RNase^2^ |
| --- | --- | --- | --- | --- | --- | --- |
| **CH 1** | 95 | 20min | 🞨 | - | 🗸 | 🞨 |
| **CH 2** | 95 | 20min | 🞨 | - | 🗸 | 🗸 |
| **CH 3** | 56 | overnight | 🗸 | 🗸 | 🗸 | 🗸 |
| **CH 4** | 56 | 4h | 🗸 | 🞨 | 🗸 | 🞨 |
| **CH 5** | 56 | 4h | 🗸 | 🗸 | 🞨 | 🞨 |
| **CH 6** | 56 | 4h | 🗸 | 🞨 | 🞨 | 🞨 |

^1^95°C, 10 min; ^2^37°C for 8 min.
