# Supplementary material for: Extracting the invisible: obtaining high quality DNA is a challenging task in small arthropods
Source: PeerJ. 2019 Apr 12;7:e6753. doi: 10.7717/peerj.6753 (PMC6463856; doi:10.7717/peerj.6753)
Supplement: Supplemental Information 2 [file peerj-07-6753-s002.docx]

**Concentration measurements (ng/µL) of DNA extracts by UV spectrometry (NanoDrop), obtained from three oribatid mite species.**

| **Extraction method** | ***Tectocepheus* sp.** | | ***Paraleius leontonychus*** | | ***Hermannia* sp.** | |
| --- | --- | --- | --- | --- | --- | --- |
| **NST** | 3.33 | 2.67 | 5.00 | 4.50* | 3.67 | 5.17 |
| **QIA** | 1.83 | 2.83* | 2.83 | 2.50* | 3.50 | 3.00 |
| **PEQ** | x | 0.50^1^ | x | 1.67 | 0.00 | x |
| **WIP** | 15.5^1^ | 31.00^1^* | 23.67^1^ | 33.00* | 27.33 | 15.50 |
| **CTAB** | 4.67 | 10.83 | 4.83 | 1.33* | x | x |
| **EZNA** | 0.67 | 1.50 | 0.33 | 1.67* | 4.16 | - |
| **CH 1** | 8.5 | 6.5* | 8.17 | 6.83* | 34.50 | 51.50 |
| **CH 2** | 2.17 | 3.17 | 4.00 | 4.33* | 17.00 | 16.17 |
| **CH 3** | 36.50 | 33.83 | 42.83 | 38.83* | 63.67 | 74.17 |

Same samples are indicated by same shades (white or grey) as found in Table 2. Values are given for single individuals (two per species). For the EZNA only one *Hermannia* individual was investigated. Values represent the mean of three replicate measurements (with new blanks).

x=value lower than blank

^1^no PCR product

*specimens preserved in propylene glycol
