# Supplementary material for: Extracting the invisible: obtaining high quality DNA is a challenging task in small arthropods
Source: PeerJ. 2019 Apr 12;7:e6753. doi: 10.7717/peerj.6753 (PMC6463856; doi:10.7717/peerj.6753)
Supplement: Supplemental Information 3 [file peerj-07-6753-s003.docx]

**Purity of DNA extracts (mean OD values for each method, highest and lowest value, SD, and number of measured samples) obtained from nine different methods.**

| **DNA extraction method** | **A260/280**** | **Range** | **SD** | **Measured samples** |
| --- | --- | --- | --- | --- |
| **NST** | 2.45 | 1.60-5.00 | 2.40 | 7 |
| **QIA** | 1.73 | 1.00-4.00 | 2.12 | 6 |
| **PEQ*** | - | - | - | - |
| **WIP** | 1.93 | 1.72-2.07 | 0.24 | 6 |
| **CTAB** | 2.85 | 2.00-5.00 | 2.12 | 6 |
| **EZNA** | 1.60 | 0.50-2.00 | 1.06 | 5 |
| **CH1** | 2.00 | 1.55-2.29 | 0.52 | 6 |
| **CH2** | 1.73 | 1.33-2.25 | 0.65 | 6 |
| **CH3** | 1.03 | 0.91-1.19 | 0.20 | 6 |

* concentration of DNA too low to measure OD.

** mean value of all samples

SD = standard deviation from independent DNA extractions
