# Supplementary figures and images for: Extracting the invisible: obtaining high quality DNA is a challenging task in small arthropods
Source: PeerJ. 2019 Apr 12;7:e6753. doi: 10.7717/peerj.6753 (PMC6463856; doi:10.7717/peerj.6753)

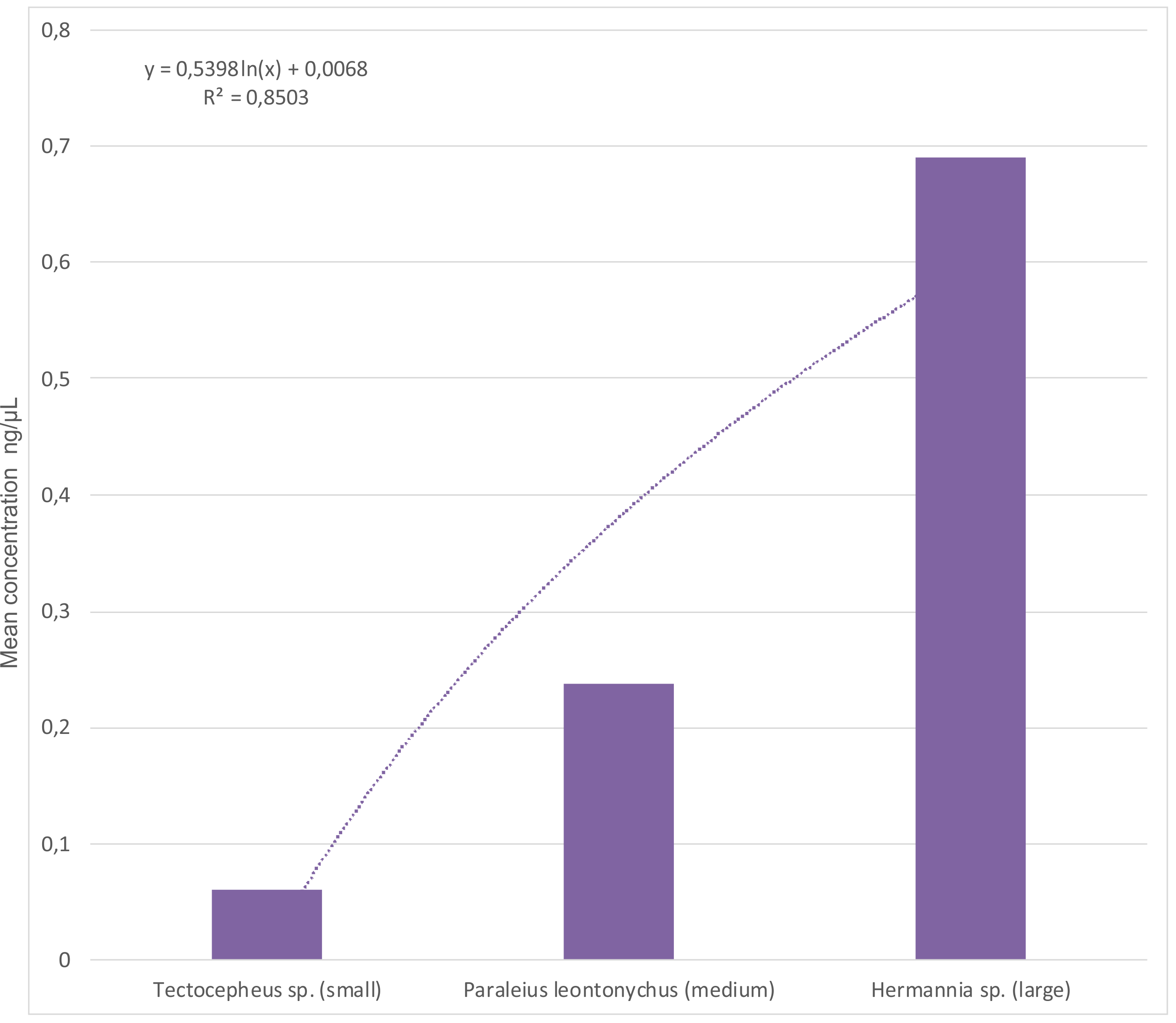

Supplement: Supplemental Information 4 [file peerj-07-6753-s004.png]

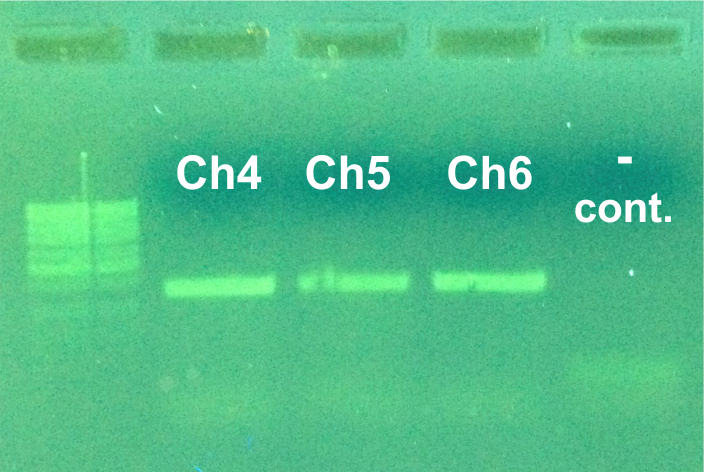

Supplement: Supplemental Information 5 [file peerj-07-6753-s005.png]

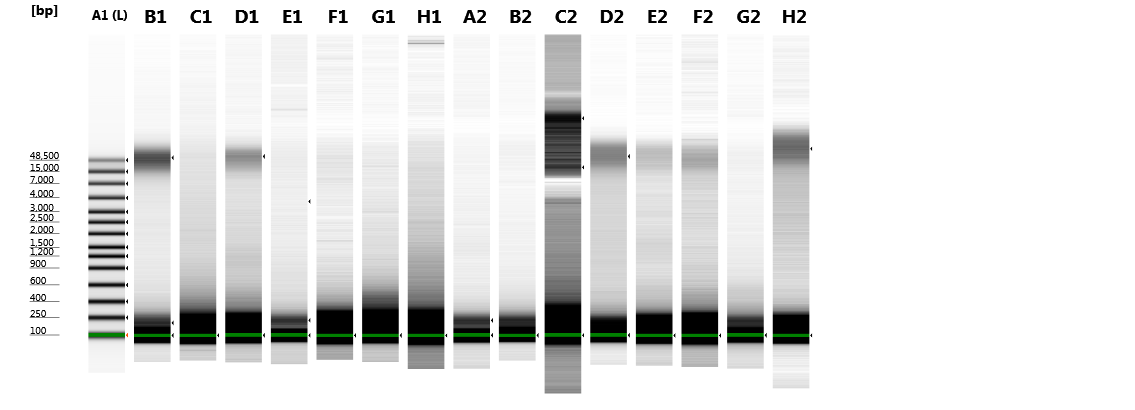

Supplement: Supplemental Information 6 — This image was combined with image Part2 in Fig. 2 of the manuscript. Not all lanes are used for the present study. [file peerj-07-6753-s006.png]

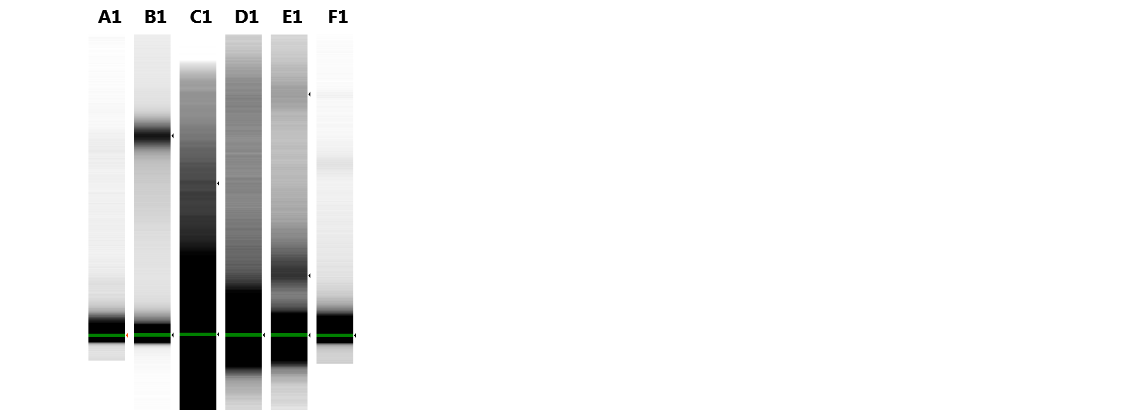

Supplement: Supplemental Information 7 [file peerj-07-6753-s007.png]
